# The assessment of a manikin-based low-dose, high-frequency cardiac resuscitation quality improvement program in early UK adopter hospitals

**DOI:** 10.1186/s41077-021-00168-y

**Published:** 2021-04-21

**Authors:** Katherine Kuyt, Montana Mullen, Catherine Fullwood, Todd P. Chang, James Fenwick, Victoria Withey, Rod McIntosh, Naomi Herz, Ralph James MacKinnon

**Affiliations:** 1 Department of Research & Innovation, Manchester University NHS Foundation Trust, Manchester, UK; 2 Medical Statistics Group, Manchester University NHS Foundation Trust, Manchester, UK; 3 Centre for Biostatistics, University of Manchester, Manchester, UK; 4 Division of Emergency Medicine and Transport, Children’s Hospital of Los Angeles, Los Angeles, USA; 5 Resuscitation Service, Basildon University Hospital, Mid and South Essex NHS Foundation Trust, Southend-on-Sea, UK; 6 Spire Cheshire Hospital, Warrington, UK; 7 Department of Resuscitation, Borders General Hospital, Borders NHS, Selkirk, UK; 8 British Heart Foundation, London, UK; 9 Department of Paediatric Anaesthesia, Royal Manchester Children’s Hospital, Manchester University NHS Foundation Trust, Manchester, UK

**Keywords:** Cardiopulmonary resuscitation, Simulation, Training

## Abstract

**Background:**

Adult and paediatric basic life support (BLS) training are often conducted via group training with an accredited instructor every 24 months. Multiple studies have demonstrated a decline in the quality of cardio-pulmonary resuscitation (CPR) performed as soon as 3-month post-training. The ‘Resuscitation Quality Improvement’ (RQI) programme is a quarterly low-dose, high-frequency training, based around the use of manikins connected to a cart providing real-time and summative feedback. We aimed to evaluate the effects of the RQI Programme on CPR psychomotor skills in UK hospitals that had adopted this as a method of BLS training, and establish whether this program leads to increased compliance in CPR training.

**Methods:**

The study took place across three adopter sites and one control site. Participants completed a baseline assessment without live feedback. Following this, participants at the adopter sites followed the RQI curriculum for adult CPR, or adult and infant CPR. The curriculum was split into quarterly training blocks, and live feedback was given on technique during the training session via the RQI cart. After following the curriculum for 12/24 months, participants completed a second assessment without live feedback.

**Results:**

At the adopter sites, there was a significant improvement in the overall score between baseline and assessment for infant ventilations (*N* = 167, *p* < 0.001), adult ventilations (*n* = 129, *p* < 0.001), infant compressions (*n* = 163, *p* < 0.001) adult compressions (*n* = 205, *p* < 0.001), and adult CPR (*n* = 249, *p* < 0.001). There was no significant improvement in the overall score for infant CPR (*n* = 206, *p* = 0.08). Data from the control site demonstrated a statistically significant improvement in mean score for adult CPR (*n* = 22, *p* = 0.02), but not for adult compressions (*N* = 18, *p* = 0.39) or ventilations (*n* = 17, *p* = 0.08). No statistically significant difference in improvement of mean scores was found between the grouped adopter sites and the control site. The effect of the duration of the RQI curriculum on CPR performance appeared to be minimal in this data set. Compliance with the RQI curriculum varied by site, one site maintained hospital compliance at 90% over a 1 year period, however compliance reduced over time at all sites.

**Conclusions:**

This data demonstrated an increased adherence with guidelines for high-quality CPR post-training with the RQI cart, for all adult and most infant measures, but not infant CPR. However, the relationship between a formalised quarterly RQI curriculum and improvements in resuscitation skills is not clear. It is also unclear whether the RQI approach is superior to the current classroom-based BLS training for CPR skill acquisition in the UK. Further research is required to establish how to optimally implement the RQI system in the UK and how to optimally improve hospital wide compliance with CPR training to improve the outcomes of in-hospital cardiac arrests.

## Background

High-quality cardiopulmonary resuscitation (CPR) is critical for patient survival during a cardiac arrest, providing manual circulation of oxygenated blood while any reversible causes of the cardiac arrest are identified and treated [1]. The national cardiac arrest audit for 2018/2019 reported the incidences of in-hospital cardiac arrest in 192 acute NHS hospitals, of which there were 14,139 in the reported period (1% of total admissions). Only 53.1% of the in-hospital cardiac arrests survived, as defined as a return of spontaneous circulation (ROSC) for greater than 20 min, of which 23.6% of patients survived until hospital discharge [2].

Training for cardiac arrests present an important and acute challenge for healthcare professionals (HCPs) and hospital organisations. High-quality CPR has been identified as the “primary component in influencing survival from cardiac arrest” [1] yet often these standards are not being met. High-quality CPR includes appropriate rate, depth, and chest recoil for adult CPR, as well as appropriate ventilation for paediatrics. It has been reported by previous studies that high-quality chest compressions do not occur in 36–87% of in-hospital CPR [3–5].

Adult and paediatric basic life support (BLS) training is most often conducted via in-person group training with an accredited instructor, termed Resuscitation Practitioners or Resuscitation Training Officers. The instructor visually assesses the CPR technique of the HCPs and decides whether or not they meet the required standard. Studies have reported that CPR training instructors can be unreliable at determining chest compression quality [6, 7]. Additionally, training is typically conducted annually/biannually, despite data demonstrating a decline in quality of CPR performed as soon as 3 months post-training [8–10]. Many HCPs do not perform CPR regularly outside of mandatory training, and therefore it is likely that if called upon to perform CPR their skills will have declined since last having completed BLS training [2]. This is the rationale for the ‘Resuscitation Quality Improvement’ (RQI) programme, a quarterly ‘low-dose, high-frequency’ training, with a competency-based approach. RQI is training based around the use of manikins that provide real-time user-feedback on CPR performance. The initial implementation of an RQI programme, by the American Heart Association and Laerdal USA (Wappinger Falls, NY), has produced promising results in terms of improving resuscitation education, training compliance, cost-saving and lifesaving potential through highly trained staff, in the USA [8, 11]. However, this low-dose, high-frequency training approach is new in the UK.

The UK healthcare system is divided into free access, publicly funded National Health Service (NHS) hospitals and ‘private’ hospitals where a charge is levied for healthcare, usually paid by medical insurance. The NHS dominates healthcare provision in the UK, with approximately 11% of the population having private medical insurance [12]. As per private hospitals, each NHS hospital is able to adopt a new training paradigm to enhance the CPR training of individuals to improve the outcome of in-house cardiac arrests and to improve the hospital wide compliance of CPR training by HCWs. Compliance of CPR training, that is the percentage of hospital staff who have completed their CPR training within the time frame set by each hospital, (usually 1 or 2 years) is one of a raft of quality metrics, by which each UK hospital is independently audited against nationally agreed standards [13]. There are financial rewards to hospitals excelling in meeting care commissioned standards [14] and punitive steps for those that are failing. In the UK, there is currently no evidence to support a change from the status quo of current BLS training, studies that demonstrate a positive impact of new training paradigms could positively influence hospital organisations to make such a change.

This study aimed to evaluate the RQI Programme at early adopter sites in the UK, to determine whether RQI training improves individual CPR psychomotor skills performance, and to establish whether the RQI program leads to increased hospital compliance with mandatory CPR training by healthcare staff.

## Methods

### Study design

This was a multi-institutional cohort study conducted at 4 UK hospitals from October 2017 to March 2020, prior to the COVID-19 pandemic in the UK. The evaluation was conducted over a 1- or 2-year period at each site. The hospitals partaking in this study consisted of one private hospital and three NHS hospitals. The control site was an NHS hospital.

### Study participants and setting

Any members of staff employed by, and working at the site, were eligible to partake in the study. This was at the discretion of the study sites, with individual sites electing which roles and departments participated in the trial. Pre-qualification students were excluded from the study.

The RQI system was physically placed in the hospital at the discretion of the site. Participants had access to the RQI manikins throughout the duration of the study; site participants were sent reminders to complete the curriculum each quarter, automatically by the RQI system.

**Fig. 1:**
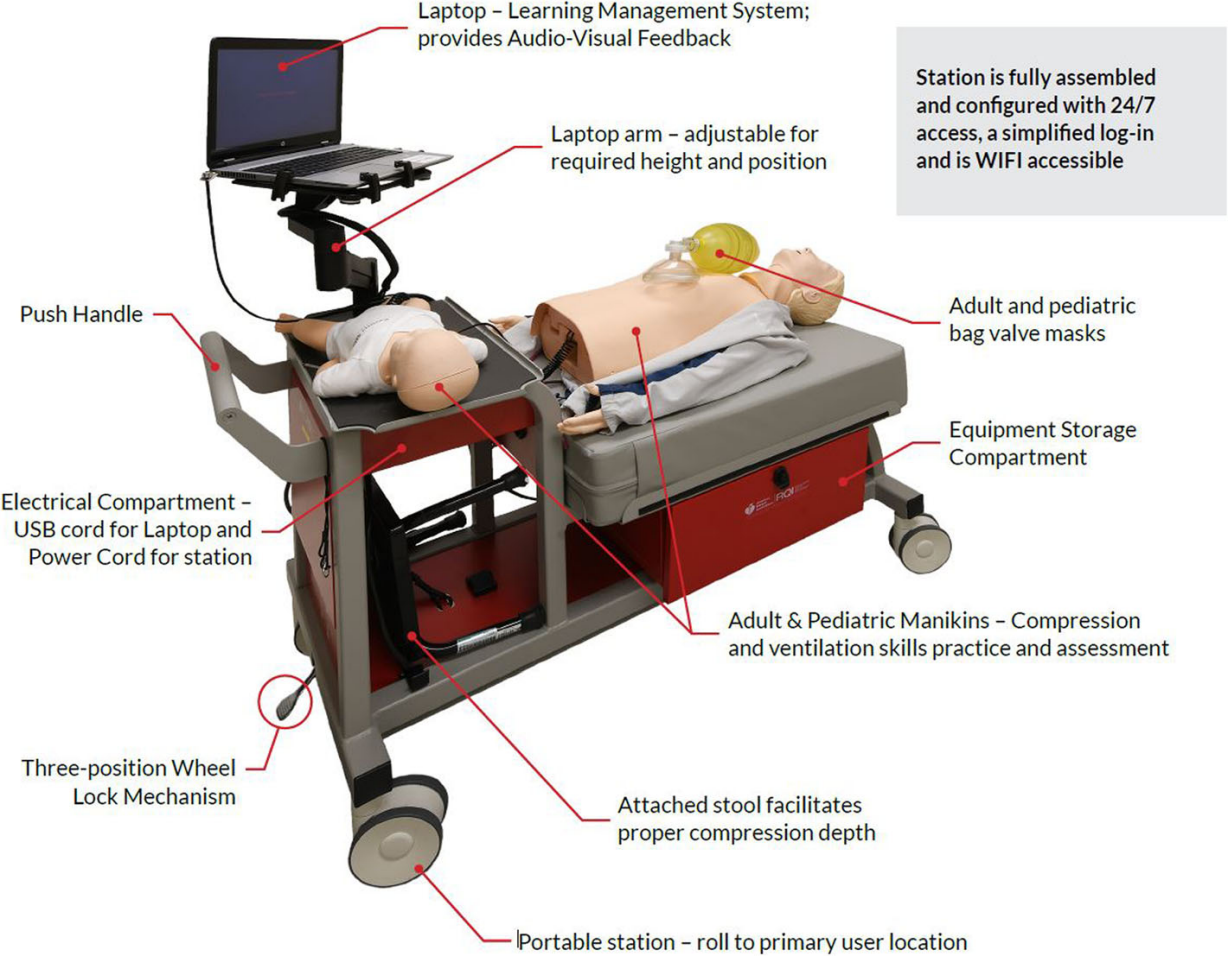
The RQI cart. Including adult and infant manikins, bag-valve mask, and integrated laptop computer. Included with permission from Laerdal Medical

**Fig. 2:**
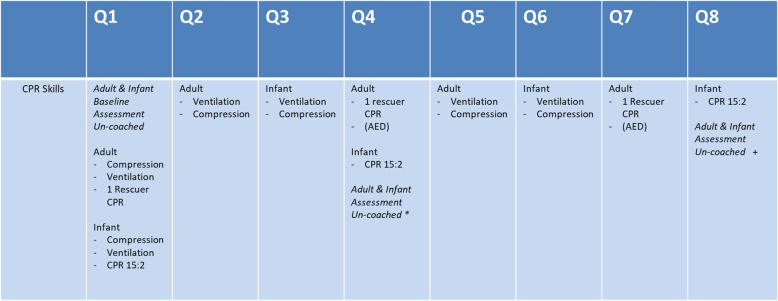
Example curriculum for a participant training in adult and infant BLS. Individuals who participated in training in adult BLS only, followed a similar structure of curriculum, but Q3, Q6, and Q8 focused on adult ventilation and adult compressions. Un-coached assessments were performed prior to commencing RQI training, and after following the curriculum for the set period of time. *Sites B and C only. + Data available for site A only

**Fig. 3:**
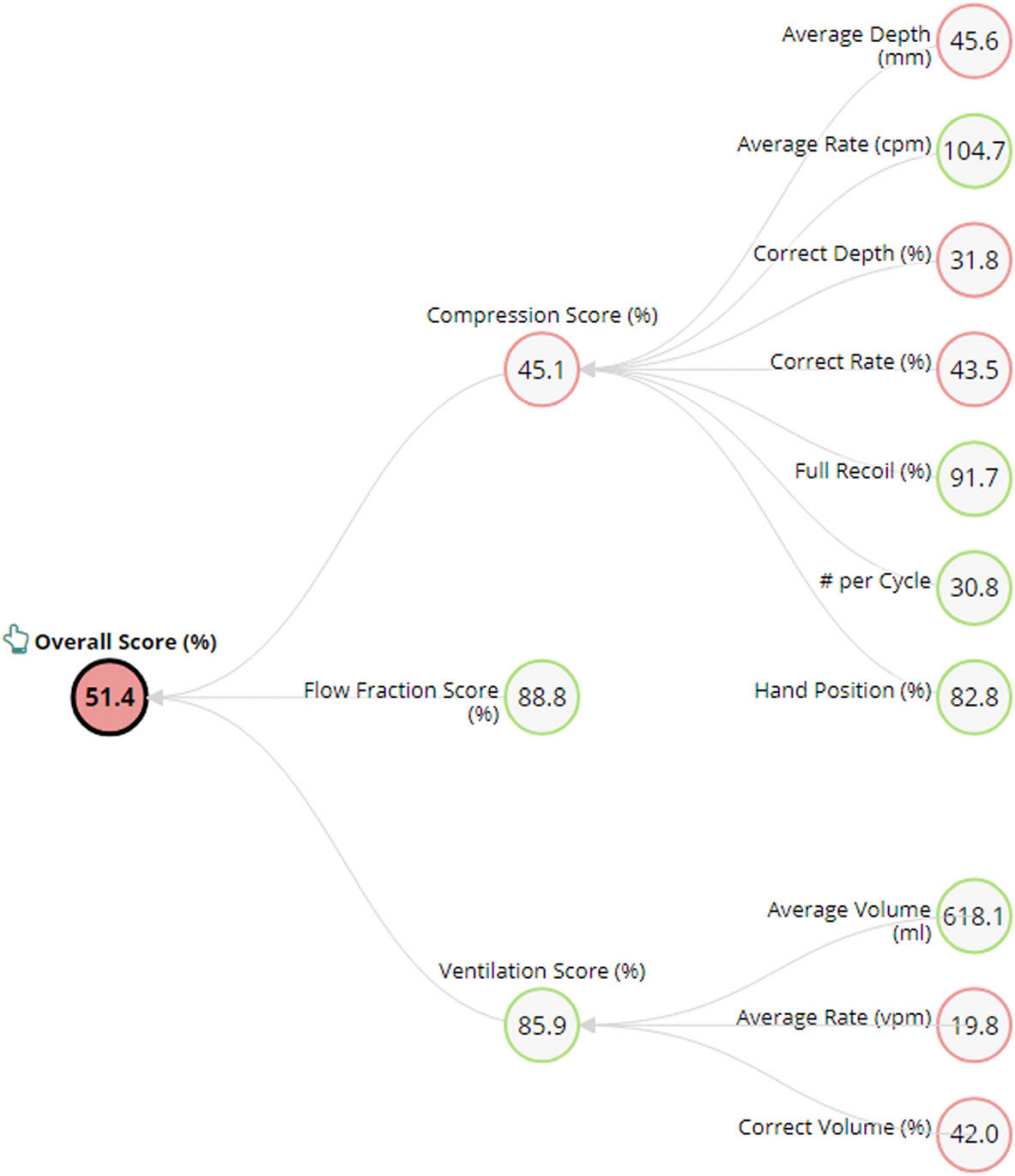
Example results and analytics from an adult rescue CPR course. Showing the overall score and the scores for different aspects that contribute. Included with permission from Laerdal Medical

The RQI cart can be seen in Fig. 1; it is composed of both adult and infant-sized QCPR manikins, and a connected tablet to display the feedback and results. The RQI curriculum divided CPR skills into 3-month blocks. As seen in Fig. 2, the foci for 3-month blocks include chest compressions, ventilations, or rescue CPR (a combination of compressions and ventilations). While practising using the RQI manikin, users receive live feedback to facilitate adjustments to their technique (such as rate and depth of compressions, and volume of ventilations) to improve performance. Upon completion of 60 compressions and/or 1 min of ventilations, RQI presents a summary screen of the session, displaying an overall performance score, and the different aspects that contribute to the score (Fig. 3).

Three hospital sites in the UK took part in this evaluation project as active sites. Each adopter site was able to enrol up to 250 participants into the RQI program to form the intervention group. UK hospitals that had purchased the RQI system were approached by Laerdal and asked whether they wished to become a site for this study. Each site was free to determine where the RQI programme would be applied to the healthcare staff in their organisation, in terms of which staff would be enrolled. The sites had further control over whether to enrol individual participants into the adult-only, infant-only, or adult and infant curriculum. The RQI system replaced the previous BLS training that the hospital provided for the participants. The research team contacted each hospital only after being notified by Laerdal that the hospital had purchased and set up the RQI system in each hospital. It was made clear to a link person at each site, a resuscitation practitioner, that the researchers were independent and not associated with Laerdal or the British Heart Foundation and only analysing the data provided by the RQI system. Also, that all data would be anonymised at the individual and hospital level. There was no contact from the research team with any participating individuals at the adopter sites throughout the study. No incentives were provided at the sites and there was no proctoring from the researchers.

Participants at each adopter site performed a baseline assessment prior to commencing the RQI program and then an assessment at either 12 or 24 months from baseline. The assessments required an individual to perform rescue CPR, (both ventilations and compressions), for 1 min, then to perform 1 min of ventilations only and 1 min of compressions only. A colleague held open the airway and the bag-mask valve, and the participant squeezed the bag to provide ventilation for the rescue CPR assessment. Participants could rest between the three assessments for as long as required to minimise fatigue. Assessments would occur on the adult, infant, or both manikins dependent on whether the hospital chose adult, infant, or both of the curricula.

The RQI device trains individuals using live feedback. No live feedback was provided for any of the baseline, 12- or 24-month assessments in order to evaluate the unguided performance of participants. The RQI cart collected the raw data which was forwarded from the propriety cloud-based system to a data hive at the primary research institution for analysis. Each interaction with RQI cart generated an anonymised 16 alphanumeric code; these were collated and analysed in quarterly cycles from the data hive for each hospital. Only the primary investigator (RM) was unblinded to the identity of the data sources, in terms of which hospital data were from. The pooled assessment data for each hospital served as the primary outcome to compare against the pre-RQI baseline data.

In addition to the active adopter sites, there was a control site, an adult hospital that had access to the RQI system without structured curricula. Participants at this site undertook a baseline assessment and then had a second assessment after 12 months (12M); however, they did not have access to the RQI cart or follow the RQI curriculum between the two assessments. No infant RQI system was placed in this site, due to the clinical area in which the cart was situated.

### Outcome measures

Data were divided into two separate domains: CPR psychomotor skills and CPR training compliance.

#### CPR psychomotor skills

The RQI system assessed participants against multiple high-quality compression and ventilation metrics measured by the manikin sensors and calculated an overall score from 0 to 100%. If being assessed for ‘rescue CPR’, then the compression and ventilation scores were combined for the final score. The score is a proprietary Laerdal calculation that includes metrics for high-quality performance in CPR. The Laerdal score was calculated based on compliance with the Resuscitation Council of the UK (RCUK) standards for compressions and ventilations. Adult RCUK standards are the following: a compression rate of 100–120 per min, a compression depth of 50–60 mm, full chest recoil between compressions, and the correct hand position. RCUK high-quality ventilation standards are a tidal volume 400–700 ml and a ventilation rate of 8 to 12 breaths/min. For infant CPR compression, depth is reduced to 40 mm. Also, the ventilation standards are a tidal volume of 20–40 mls, with a ventilation rate of 12–20 breaths/min.

Participants were required to score a minimum of 75% to pass the course each quarter, and the learning module to be classed as ‘completed’. However, participants were not restricted upon the number of attempts they were able to make to reach 75%, nor were they prevented from continuing to practise on the RQI once they had passed the curriculum.

#### Compliance

The compliance for each participant was measured as attendance data. A participant was deemed compliant if the RQI system logged full participation with the curriculum within the 3-month quarter; that is an individual had completed and received a passing score in all the courses allocated by the RQI system within the quarter. Courses were either classed as completed, in progress, or not started; only courses listed as complete were classed as compliant. Participants could be compliant in one course and non-compliant in another course throughout the study period, which yields a % compliance. Compliance was assessed quarterly from the study set up to Q4 2019.

### Data analysis

Every effort was made to ensure all data was unfiltered and sent directly to the research server at the researcher’s site and only analysed by the research team. All data analysis was performed by authors who were blinded to the site identity [KK, TC, CF], to prevent bias in the analysis. This evaluation was based upon an opportunistic sample, 3 active samples, and 1 control recruiting up to 250 participants would provide a broad range of staff levels and type accessing either infant or adult training.

Descriptive statistics (mean and standard error of the mean) were used to summarise CPR psychomotor skills at baseline and assessment for adopter sites and the control site. Paired *t* tests were used to test for improvement from baseline to assessment for both the control site and the grouped adopter sites. An additional unpaired *t* test was performed to assess for a difference between the changes in the score at the 3 pooled adopter sites vs. the 1 control site. Two-tailed *t* tests were also performed to compare the baseline scores for the control sites and adopter sites.

Further analyses were undertaken to determine whether the length of following the RQI curriculum (4 quarters versus 8 quarters) led to significant differences in the magnitude of improvement of skill scores. Data was grouped for the two adopter sites who performed the assessment after 4 quarters.

Alpha was set at 0.05 for all analyses, in line with standard practice. Analyses were completed using Microsoft Excel (Microsoft Office 2010, USA) and R v3.6.0 (R Core Team, 2019).

## Results

### Participant population

Data collection began in October 2017 and was stopped at the end of March 2020 due to the COVID-19 pandemic. Participants continued to enter the study at quarterly intervals throughout the study duration. Data collection stopped with a total of 1861 participants enrolled on the RQI system, 487 at the control site, and 1374 from across the adopter sites. Paired baseline and assessment data was available for a total of 321 participants from across the study sites. Of these participants, 249 had completed the assessment in adult CPR, and 206 had completed the assessment in infant CPR.

One site (site B) had enrolled a range of staff across the entire hospital clinical and non-clinical, including administration and management staff. The other active sites enrolled only healthcare professionals that may be expected to perform clinical CPR and were required to certify or re-certify for BLS. These included physicians, nurses, physiotherapists, and pharmacists, among others.

### CPR psychomotor skills

#### CPR performance at adopter sites

**Fig. 4:**
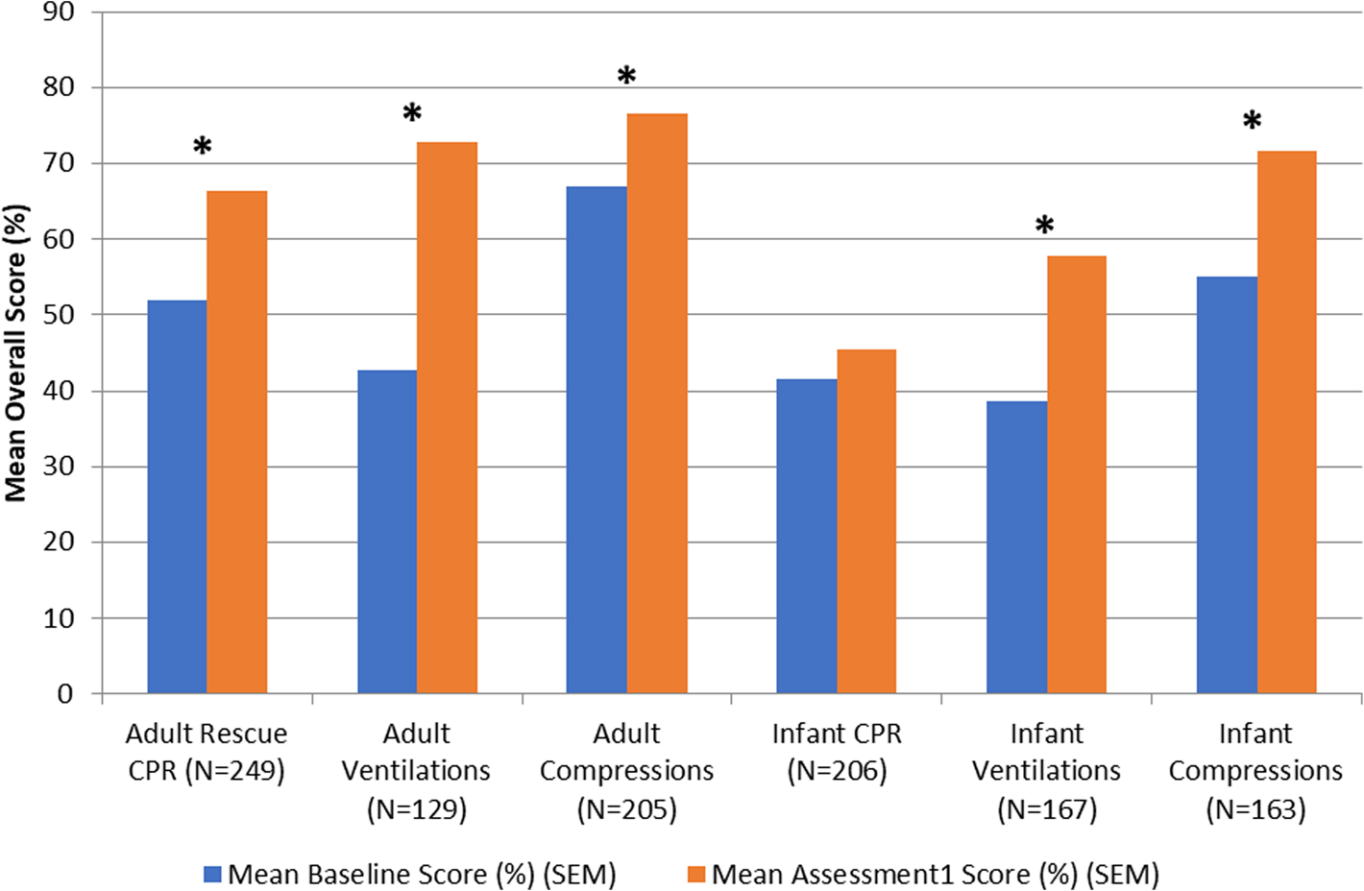
Mean baseline and assessment scores for adopter sites. Mean scores, data grouped across all three adopter sides. *p < 0.001

Data from the three adopter sites were grouped for primary analysis, and scores < 1% were classed as outliers and removed in a pairwise manner (45 adult and 79 infants). There was a significant improvement (*p* < 0.001) in the overall score between baseline and assessment for; infant and adult ventilations, infant and adult compressions, and adult CPR. There was no significant improvement in the overall score for infant CPR (Table 1, Fig. 4).

**Table 1:** Mean scores at baseline and assessment 1 for the six un-coached assignments. Only paired data used. Mean Difference = the difference in mean baseline score and mean assessment score. *p* value obtained from a paired *t* test

CPR performance	Mean baseline score (%) (SEM)	Mean assessment score (%) (SEM)	Mean difference (95% CI)	***p*** value
Adult rescue CPR (*n* = 249)	51.9 (1.6)	66.4 (1.5)	14.4 (10.7–18.2)	< 0.001
Adult ventilations (*n* = 129)	45.8 (3.1)	72.8 (2.8)	26.9 (20.0–33.9)	< 0.001
Adult compressions (*n* = 205)	67.0 (2.1)	76.5 (1.9)	9.5 (4.0–15.0)	< 0.001
Infant 15:2 CPR (*n* = 206)	41.6 (1.6)	45.4 (1.7)	3.8 (− 0.5–8.0)	0.08
Infant ventilations (*n* = 167)	38.7 (2.3)	57.8 (2.1)	19.1 (13.5–24.7)	< 0.001
Infant compressions (*n* = 163)	55.0 (2.7)	71.6 (2.6)	16.5 (9.1–23.9)	< 0.001

#### CPR performance at control site

Data from the control site demonstrated a statistically significant improvement in the mean score for adult CPR (*p* = 0.021), but not for adult compressions or ventilations (Table 2).

**Table 2:** Mean baseline and assessment scores for the control site. Mean score and standard error of the mean. *p* value from a two-tailed paired *t* test

CPR performance	Mean baseline score (%) (SEM)	Mean assessment score (%) (SEM)	Mean difference (95% CI)	***p*** value
Adult rescue CPR (*n* = 22)	49.2 (5.5)	64.3 (5.2)	15.1 (2.5–27.8)	0.021
Adult ventilations (*n* = 17)	59.9 (8.1)	79.8 (7.8)	19.9 (− 2.6–42.5)	0.079
Adult compressions (*n* = 18)	57.8 (6.0)	63.7 (7.0)	5.9 (− 8.2–20.0)	0.390

The mean time between baseline and assessment for participants in the control group was 332.9 days (range 160–526 days, SD 96.7 days). There was a moderate negative correlation between time eclipsed from the baseline to assessment, and change in the overall score for adult CPR (*R* = − 0.44, *p* = 0.04), however, no such correlation was seen for adult compressions or ventilations.

#### Comparison of CPR performance between adopter and control sites

There was no significant difference between baseline scores at the control and adopter sites for adult CPR (*p* = 0.62), compressions (*p* = 0.21) or ventilations (*p* = 0.12). Table 3 shows the mean improvement in the overall score for the adult assessments compared between adopter sites and the control site. No significant differences in mean improvement were found between the control site and the grouped adopter sites.

**Table 3:** The mean improvement in overall scores between baseline and assessment for the control site and adopter sites. Data tested for a difference between the groups via two-tailed, unpaired *t* tests. Adopter site data is grouped regardless of time point

Study sites	Mean improvement CPR (%) (SEM)	Mean improvement compressions (%) (SEM)	Mean improvement ventilation (%) (SEM)
Control site	15.1 (6.1) (*n* = 22)	5.9 (10.6) (*n* = 18)	19.9 (10.6) (*n* = 17)
Adopter sites	14.5 (1.9) (*n* = 249)	9.5 (2.8) (*n* = 205)	27.0 (3.5) (*n* = 129)
*p* value	0.918	0.622	0.537

### The effect of duration of the RQI curriculum on CPR performance

Table 4 details the overall score data split according to whether the assessment was performed after 4 quarters or 8 quarters of following the RQI curriculum. Except for adult ventilation, no significant differences were found between the mean improvement in the scores of the two groups.

**Table 4:** The mean improvement in overall scores between baseline and assessment for the participants performing A_a_ and participants undergoing A_b_

RQI curriculum	4 quarter assessment mean improvement (%) (SEM)	8 quarter assessment mean improvement (%) (SEM)	Mean difference (95% CI)	***p*** value
Adult rescue CPR	17.1 (2.4) (*n* = 118)	12.1 (2.9) (*n* = 131)	− 4.9 (− 12.5–2.6)	0.191
Adult ventilations	18.5 (4.9) (*n* = 62)	34.8 (4.8) (*n* = 67)	16.2 (2.6–29.9)	0.020
Adult compressions	15.0 (3.8) (*n* = 99)	4.4 (4.0) (*n* = 106)	− 10.5 (− 21.4–0.4)	0.058
Infant CPR	1.7 (2.9) (*n* = 89)	5.4 (3.1) (*n* = 117)	− 3.7 (− 12.3–4.9)	0.382
Infant ventilations	15.6 (4.4) (*n* = 81)	21.0 (3.7) (*n* = 86)	5.5 (− 5.9–16.9)	0.342
Infant compressions	17.2 (5.3) (*n* = 79)	15.9 (5.3) (*n* = 84)	− 1.4 (− 16.2–13.4)	0.853

***A***_***a***_ assessment occurred after 4 quarters of following the RQI curriculum, ***A***_***b***_ assessment occurred after 8 quarters of following the RQI curriculum. Data tested for differences between the groups (***p*** < 0.05) via two-tailed, unpaired ***t*** tests

**Fig. 5:**
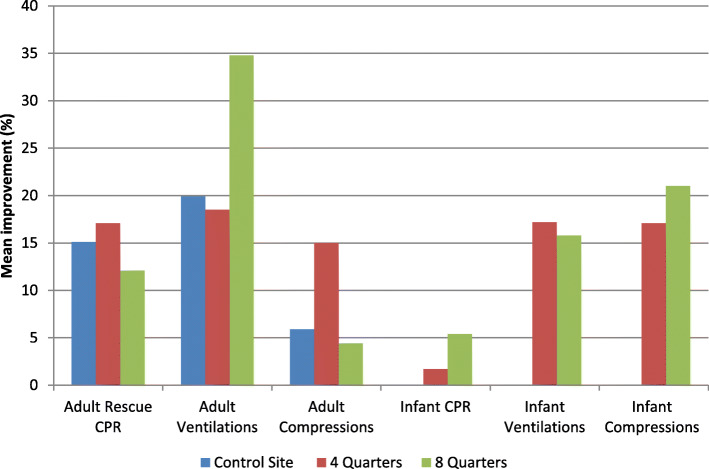
Mean improvement in overall score from baseline to assessment scores according to the length of the curriculum followed. Data spilt into control site (no curriculum), sites assessed after following the curriculum for 4 quarters, and the site assessed after following the curriculum for 8 quarters. The control site did not partake in infant assessments

Figure 5 displays the mean improvement from baseline to assessment for the three groups: control site (no curriculum), 4 quarters of curriculum, and 8 quarters of curriculum. This further illustrates that there is no linear correlation between the length of the RQI curriculum and improvement in score.

### Compliance

**Fig. 6:**
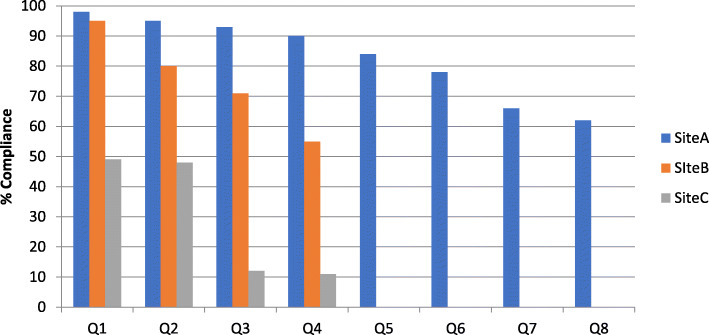
Overall compliance during the RQI curriculum, by site. Each participant could be assigned more than one course per quarter; for example, adult ventilations and adult compressions. Assessment performed after Q4 for sites B and C

Compliance data are presented for all 1374 participants that entered the RQI program. Compliance is reported as the percentage of individuals in the study who had completed the courses allocated to them by the RQI system at their respective hospital sites. The compliance reduced over time at all sites. Figure 6 displays the percentage of courses completed per quarter for the three sites that followed the assigned RQI curriculum for a minimum of 12 months (four quarters). Site A had the best overall compliance with 98% of courses being completed in the first quarter, only reducing to 90% of assigned courses completed in the fourth quarter. Whereas, at site C, only 49% of the courses assigned in the first quarter were completed, and compliance reduced subsequently each quarter. Site A was the site that chose to assess the CPR skill scores after eight quarters of the curriculum, rather than four. During quarters five to eight the overall compliance of assigned courses was 84%, 78%, 66%, and 62%, respectively.

**Fig. 7:**
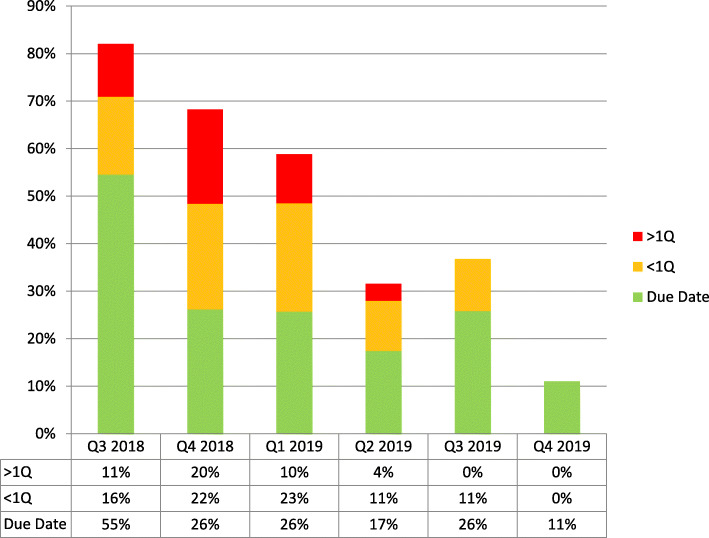
Percentage compliance according to due date across all sites. The percentage of assigned courses completed by due date, completed less than one quarter past due, and completed more than one-quarter past due. Data grouped across all sites according to the due date, regardless of curriculum stage assigned

A further measure of compliance is those courses completed in the scheduled quarter. Figure 7 illustrates compliance according to the course due date, for all adopter sites, showing data for only the courses due during the assigned quarter, not cumulative figures. Except for Q3 2019, the percentage of incomplete studies increases with each quarter and the percentage of courses completed by their due date, at the end of the assigned quarter, fall continually over time. The rates of courses being completed, but past the due date, stays comparatively stable.

## Discussion

We present data from the first multi-centre implementation of a novel mode of learning CPR in UK hospitals. In this evaluation, participation in the RQI program improved individual’s CPR psychomotor skills performance on both an adult and infant manikin. The overall scores improved between baseline and assessment for adult CPR, compressions, and ventilations. There was also an improvement for infant ventilation and compressions but not for infant CPR. There was no significant difference in improvement when comparing the control site that did not participate in the RQI curriculum and the group of adopter sites. In this study, compliance decreased steadily across the duration of the study, albeit from a position of high compliance in two of the adopter sites.

High-quality compression is known to improve survival rates and neurological recovery among patients in cardiac arrest [15]. In this UK study, the baseline scores of CPR psychomotor skills of the participants using the RQI cart showed room for improvement, and after a 1- or 2-year curriculum, participants improved their CPR psychomotor skills. This aligns with previous work that reports on the positive impacts of the RQI cart in the USA [8, 11]. Live, objective, feedback during training sessions, along with a feedback report immediately afterwards are key features of the RQI cart. Previous work has demonstrated the positive effects of feedback during CPR, regardless of if it is being performed in-hospital or out of hospital [16, 17]. An effective method that allows individuals to focus upon their own CPR psychomotor skills alone with a feedback device has the potential to change mandatory basic life support training in the UK. If individuals could maintain their CPR performance levels quarter by quarter and prevent the known CPR skill decay [8–10], this could potentially minimise the time spent currently in classrooms that focuses purely on BLS technique. This could then provide more time to focus upon the non-technical skill training in terms of team-working and in particular communication, which have been established as a key component of optimal CPR delivery [18, 19].

The quality of infant CPR affects vital end organ perfusion, survival, and neurological outcomes [20–22]. In this study, performance scores improved for infant compressions and infant ventilation when performed independently. Chest compressions are crucial for generating perfusion pressures for vital organs [20–22]. In this study, there was no significant improvement in the infant 15:2 CPR, where the two skills of compression and ventilation were done in conjunction. This may be reflective of the fact that this does not fully represent the clinical environment; where it is likely there are two HCP’s performing CPR. Two-person CPR removes the need to alternate between compressions to ventilation in rapid succession, known to be potential difficult in infant CPR [20–22]. Further research is required to establish the effectiveness of the RQI cart for multi-person infant CPR.

This study provided an insight into how the length of a curriculum may contribute to improvement in CPR skills. It may have been expected that participants who had completed eight quarters of the RQI curriculum would outperform those who participated in four quarters. This did not occur and is suggestive that a four-quarter/yearly approach may be optimal. Only one site in this study provided data after eight quarters of the curriculum; as such site-specific factors should be taken into consideration as a potential source of bias. At each site, the RQI programme was implemented as standard practice by the local teams with no influence from researchers. Local implementation, compliance, and human motivation could directly influence the data [23]. The RQI curriculum does not have a built-in assessment at the end of each curriculum cycle; this was added for the purpose of this evaluation. The RQI curriculum is in essence formative in nature, and further research is required to determine whether a formal summative assessment stage of the curriculum may enhance learning.

Interestingly, in this study, there were also improvements in CPR psychomotor skills at the control site. Participants at the control site provided a baseline score and a yearly final assessment, with no exposure to the curriculum. The control site was an adult patient hospital, and no infant CPR was performed. It is possible that the absence of a second set of items to be tested on (infant CPR items) may have benefitted the control scores. The mean time between the baseline and assessment at the control site was 332.9 days; therefore, any feedback given on the RQI screen at the end of the baseline session is unlikely to have had a direct impact on performance in the assessments. As demonstrated by the wide confidence interval, the smaller sample size at the control site limited the power to find a statistical difference between baseline and assessment values at this site. The small sample size at the control site was in part due to difficulty in completing sufficient numbers of assessments after 12 months, with no incentives (to match the lack of incentives given at the adopter sites) and no proctoring by the research team, as per the adopter sites. Furthermore, it was logistically challenging to find willing hospitals to provide control data and or delay the implementation of a full RQI programme to provide this. As such, we are limited in our study conclusions due to a smaller control group and the heterogeneity of implementation of sites. While there were both NHS and private hospitals participating, our institution sample size is insufficient to provide insight into any system-level differences that would explain our results. The aim of this study was to determine whether participation in the RQI curriculum improves CPR performance (in hospitals that have purchased the system); we accept that a larger, suitably powered randomised controlled trial is required in order to answer the question of whether the RQI system is better or not at enhancing CPR performance than current classroom approaches in the UK.

Compliance of CPR training at a hospital level is another important factor to consider when considering changing a CPR training paradigm. A decline in RQI course user compliance was anticipated and was apparent in this study. Site A had the highest levels of compliance throughout the study, achieving a better level of compliance during quarter eight than sites B or C did in quarter four. The work of Dudzik et al., studying the implementation of the RQI system in the USA, also reported similar levels of compliance; with 56.1% of participants being fully compliant after 12 months [11]. In this study, site A sustained a compliance of 90% after 12 months and was at 60% at 24 months. Although there is no published data on this, discussion with the resuscitation department in this UK major teaching hospital indicated that compliance above 75% is strived for. In the UK, compliance with basic life support training is key hospital performance indicator measurement that is audited by an external regulator body against set standards [13, 14]. Compliance with CPR training is a metric that is focused upon at an executive board level; therefore, the impact of any training paradigm that influences this measure positively or negatively would likely be on interest to the hospital management.

Currently in the UK, staff overdue for their basic life support training are contacted by a member of the resuscitation training team, usually by email, and lack of response can be escalated through line managers. The RQI programme aims to remove the need for this administration as users have a unique user ID and are automatically emailed reminders each quarter by the RQI system to remind them to complete the assigned courses. This approach aims to place direct accountability to complete the CPR training on the learner and their responsiveness to respond to email reminders rather than direct contact by an administrator or line manager. However, as compliance is a key hospital performance measure, a degree of oversight is likely to be required. The variability in compliance across the study sites in this study suggests that leadership and external factors have a significant impact on the levels of compliance with the curriculum. As an example, post hoc analysis revealed a site with a leader who personally championed the RQI program. This member of staff continued to be involved with the RQI programme despite changing roles within the hospital.

This study has highlighted the need for further research to understand how to optimally introduce a new paradigm to learning CPR in UK hospitals. An approach to acquiring CPR skills which takes learners out of the classroom and provides opportunities to learn in the workplace has both advantages and disadvantages dependent on one’s perspective. A vital perspective to consider for future studies is that of the current providers of CPR training, namely resuscitation practitioners/resuscitation training officers in the UK. A system that could be perceived to replace face to face BLS skill teaching, that is both enjoyable and a significant part of one’s job, could be interpreted as a threat as opposed to tool that allows focus on other teaching goals, such as improved teamworking. The successful implementation of a new way of learning and maintaining CPR skills is dependent on many factors and drivers. This study has been curtailed by the COVID-19 pandemic, which in turn has significantly affected how CPR training is achieved, with an emphasis on social distancing and bag-mask ventilation rather than mouth to pocket mask. It would appear that a learning methodology centred on the individual learner, providing feedback and learning goals may be a useful way forward during these and potentially uncertain future times. It is clear from this study that a larger study that focuses on how to successfully implement a promising approach to learning the basic elements of CPR training to positively impact training compliance and improve outcomes of in-house cardiac arrests is required.

## Conclusions

These preliminary results serve to illustrate that use of the RQI system leads to increased adherence to RCUK guidelines for good quality CPR when performing simulated adult CPR, ventilations, and compressions. This is also apparent for infant ventilations and compressions. However, it is unclear whether the RQI approach is superior to the current classroom based BLS training for CPR skill acquisition in the UK. The relationship between a formalised quarterly RQI curriculum and improvements in resuscitation skills is also not clear, as skills do not appear to increase linearly as time following the RQI curriculum increases. Understanding how to enhance the compliance of staff with CPR training remains a challenge for healthcare organisations and researchers.

## Data Availability

The datasets used and/or analysed during the current study are available from the corresponding author on reasonable request.

## Abbreviations

CPR: Cardio-pulmonary resuscitation
RQI: Resuscitation Quality Improvement
ROSC: Return Of Spontaneous Circulation
NCAA: National Cardiac Arrest Audit
HCPs: Health care professionals
BLS: Basic life support
NHS: National Health Service
RCUK: Resuscitation Council of the UK
